# The Importance of Direct Patient Reporting of Adverse Drug Reactions in the Safety Monitoring Process

**DOI:** 10.3390/ijerph19010413

**Published:** 2021-12-31

**Authors:** Kamila Sienkiewicz, Monika Burzyńska, Izabela Rydlewska-Liszkowska, Jacek Sienkiewicz, Ewelina Gaszyńska

**Affiliations:** 1 Department of Management and Logistics in Healthcare, Medical University of Lodz, Lindleya Street 6, 90-131 Lodz, Poland; izabela.rydlewska-liszkowska@umed.lodz.pl (I.R.-L.); jacek.sienkiewicz@stud.umed.lodz.pl (J.S.); 2 Department of Epidemiology and Biostatistics, Medical University of Lodz, Żeligowskiego Street 7/9, 90-752 Lodz, Poland; monika.burzynska@umed.lodz.pl; 3 Department of Nutrition and Epidemiology, Medical University of Lodz, Żeligowskiego Street 7/9, 90-752 Lodz, Poland; ewelina.gaszynska@umed.lodz.pl

**Keywords:** adverse reaction, patient reporting, EudraVigilance, pharmacovigilance, safety monitoring

## Abstract

All medicinal products authorized in the European Union are subjects of constant drug-safety monitoring processes. It is organized in a pharmacovigilance system that is designed to protect human health and life by the detection, analysis and prevention of adverse drug reactions (ADRs) and other drug-related problems. The main role of the aforementioned system is to collect and analyze adverse drug reaction reports. Legislation introduced several years ago allowed patients, their legal representatives and caregivers to report adverse drug reactions, which caused them to be an additional source of safety data. This paper presents the analysis of EudraVigilance data related to adverse drug reactions provided by patients, their representatives, as well as those obtained from healthcare professionals related to medicines which belong to M01A anti-inflammatory and antirheumatic products, a non-steroid group. The objective of the study was to identify the changes in the number and structure of adverse reaction reporting after the introduction of pharmacovigilance (PV) obligations in EU. A review of scientific literature was also conducted to assess the differences in adverse reactions reported by patients or their representatives and by healthcare professionals. We also identified other factors which, according to literature review, influenced the number of adverse reaction reports provided by patients. Analysis of data collected from the EudraVigilance showed that from 2011 to 2013 the number of reports made by patients and their caregivers increased by approx. 24 percentage points, and then, from 2014, it constituted around 30% of the total of reported reactions every year, so patient reporting is an important part of pharmacovigilance system and a source of drugs’ safety information throughout their use in healthcare practice. Additionally, there was no interrelationship between the seriousness of reported adverse reactions and the overall number of patient reports when compared to reports form healthcare professionals.

## 1. Introduction

The data on the safety of medicinal products application provided by a Marketing Authorization Holder (MAH) while obtaining the first marketing authorization come mainly from preclinical and clinical trials. However, the data obtained in clinical trials are still limited owing to, among others, the sample size and lack of data collected from specific populations (e.g., pregnant women, seniors, renal or hepatic failure patients). After having been authorized in the European Union, each medication becomes subject to constant monitoring and safety assessments of its use. It is organized as a pharmacovigilance system that is designed to protect human life and health by the detection, analysis and minimalization of adverse drug reactions and other drug-related problems [1,2].

The legislation of the European Union precisely specified principles of the pharmacovigilance system, according to which both national regulatory authorities and MAH have a number of legal tasks and obligations the purpose of which is to monitor the safety of medicinal products and to detect any changes in the benefit-risk balance of the authorized medicinal products. Pharmacovigilance activities include the management of the safety of medicinal products throughout its life cycle [3,4,5].

What constitutes an important part of pharmacovigilance is the collection and analysis of individual reports of adverse reactions [6]. Legislative amendments introduced several years ago allowed patients, their legal representatives and caregivers to report adverse drug reactions, which caused the people themselves to become an additional source of information on the safety of a medicinal product after its introduction into the market.

The pharmacovigilance system is also intended to prevent adverse events which occur as a consequence of medication use. In such cases, whether the medicinal product is used in accordance with indications is of no importance. The goals of the system are achieved by the promotion of safe and effective use of medicinal products as well as a constant update of the information on the product safety [5]. Currently, pharmacovigilance activities are elements of other disciplines of research and development, which leads to a growing interest of populations in the obtained results in this field [7]. Over the past decades, the assessment of the safety of medications and their benefits has been profoundly modified by the creation of big databases and statistical programs, which has allowed for a better use of collected data and their faster analyses [8].

Adverse drug reactions are defined as “noxious and unintended responses to a medicine” [4]. They occur as a consequence of medication use in accordance with the indications as well as contrary to its registered purpose, e.g., as a result of overdose, abuse, off-label use or medical malpractice. Each case of a potential connection of an observed adverse reaction with medicinal product application is regarded as a suspected adverse reaction to a medicinal product. It is serious when at any dose the medicine use results in death, is life-threatening, requires inpatient hospitalization or the prolongation of existing hospitalization, results in persistent or significant disability or incapacity or is a congenital anomaly/birth defect [4,9,10].

All reported cases of suspected adverse reactions to medications authorized in the European Economic Area (EEA) are reported to the EudraVigilance database [11]: an electronic system designed to collect and analyze the data on medicinal products safety [12].

The person reporting the adverse event becomes the source of data on the safety of medicinal products. This could be a healthcare professional (HP) defined as a person with medical qualifications (e.g., a physician, a dentist, a pharmacist or a nurse). A consumer, defined as a non-healthcare professional (non-HP) (a patient, a patient’s relative or caregiver) is now also considered as a source of information on the safety of a medicinal product [5].

In order to prove the occurrence of an adverse reaction and its connection to the application of a medicinal product, the consumer can provide their medical records indicating such a suspicion issued by healthcare professionals (e.g., laboratory tests or other medical data). When the report is made by a patient’s medically-qualified relative, friend or caregiver, the reported information is also regarded as medically valid [5].

A drug safety profile is established through a case-by-case analysis and summary report analyses concerning the safety of medicinal products. The identification of risk and changes to the drug safety profile is based on the detection and analyses of the signals. The efficient communication of the adverse reaction reports and the observation of the changing trends is of key importance in this process [13].

“Signal is an information arising from one or multiple sources, including observations and experiments, which suggests a new potentially causal association, or a new aspect of a known association between an intervention and an event or a set of related events, either adverse or beneficial, that is judged to be of sufficient likelihood to justify verificatory action” [3]. Such information may imply a new potential relationship or a new aspect of a known adverse reaction to medicinal product. Signals management is one of crucial processes of pharmacovigilance. Single reports of adverse events also constitute a significant source of new signals. Important issues to be considered at signal detection and analysis include: prior awareness of their existence, evidence of their relationship with the application of medicinal product and their significance in clinical practice [14].

Adverse drug reactions which lead to patients’ hospitalization or prolonged hospitalization are a serious cause of clinical costs. ADRs have negative consequences not only in reference to patients’ health, but they also generate costs for the entire healthcare system [15]. Available studies concerning the costs incurred due to adverse drug reactions show that they are a cause of 3–7% of hospitalizations. The problem of achieving balance between costs and benefits of drug therapy and weighting up the costs of ADRs to the costs of avoiding them is widely discussed in scientific literature [16]. Observational studies used in PV are appropriate to assess adverse drug events and their costs and are more useful than experimental ones, even though they are more prone to bias. Systematic reviews of observational studies show that they always evaluate direct costs of ADEs, but only 7% of them also mention indirect costs [15]. Adverse effects were described as the reason for a longer hospitalization by an average of 1.2–3.8 days, with additional hospital costs of USD 2284–5640 per patient. Preventable adverse effects are between 43.3% and 80% of all adverse outcomes which lead to emergency visits and hospital admissions and are described as a reason of increased healthcare costs. The USA data analysis showed that in the year 2000, the cost of drug-related problems linked to ambulatory setting drug therapies exceeded USD 177 billion. Nonsteroidal anti-inflammatory drugs (NSAIDs) are the drugs which are widely used and are one of the leading reasons of adverse effects [17]. It is all the more important due to the fact that approx. 70% of people aged 65 years or older use at least one of the nonsteroidal anti-inflammatory drugs per week, but about 50% of them take at least 7 doses per week. This is likely to increase as the population continues to age [18].

Pharmacovigilance aims at the quickest possible identification of signals of medicinal product safety, which enables steps to be taken towards the minimalization of the consequences of potential adverse reactions [19].The collection and analysis of individual reports on adverse reactions is both the cheapest and the simplest way of drug safety assessment and new signal detection [10].

The legislation on pharmacovigilance which was adopted in 2010 and entered into force in July 2012 empowered non-HP European citizens to report any suspected adverse reactions and required national competent authorities (NCAs) and MAHs to report adverse reactions received also from, i.a., patients and their caregivers.

The aim of the changes to the legislation on medicinal product safety monitoring system in EU was to enhance the patient’s safety via monitoring, identification of new drug-related risks and risk reduction.

Since November 2017 in the EU, marketing authorization holders and national competent authorities have also been obligated to report to EudraVigilance both serious and non-serious adverse reactions to medicinal products. It is the latest and also the most vital amendment that could have affected the total number of reported cases collected in the EudraVigilance database.

This paper is a two-phase study: a review of scientific literature and an analysis of queried EudraVigilance data.

A review of scientific literature from the PubMed database was conducted to assess the differences (e.g., rates of serious adverse drug reactions, the type and number of reported reactions, completeness of reports) in adverse reactions provided by patients or their representatives and the ones reported by healthcare professionals. We have also identified other factors which, according to the literature review, have an influence on the number of adverse reaction reports provided by patients.

The aim of the study was to identify the changes in the number and structure of adverse reaction reports after the introduction of pharmacovigilance obligations in the EU.

The specific objectives of this part of the study were:

1. To evaluate changes in the number of reports submitted by consumers and healthcare professionals between 2011 and 2020 for substances belonging to M01A group-anti-inflammatory and antirheumatic products, non-steroids group.
2. To evaluate how the proportion of patient’s reports changed after the introduction of EU PV obligations for the analyzed substances.
3. To analyze differences in the structure of adverse drug reaction reports for analyzed substances.

Research questions defined for this part of study:

Under-reporting is a major limitation of the pharmacovigilance system. Has the introduction of the patient as a source of adverse drug reactions reports increased the overall number of collected reports?

What is the structure of collected adverse reactions reports by seriousness and reporter group with regard to the introduced legislative changes?

## 2. Materials and Methods

**Figure 1:**
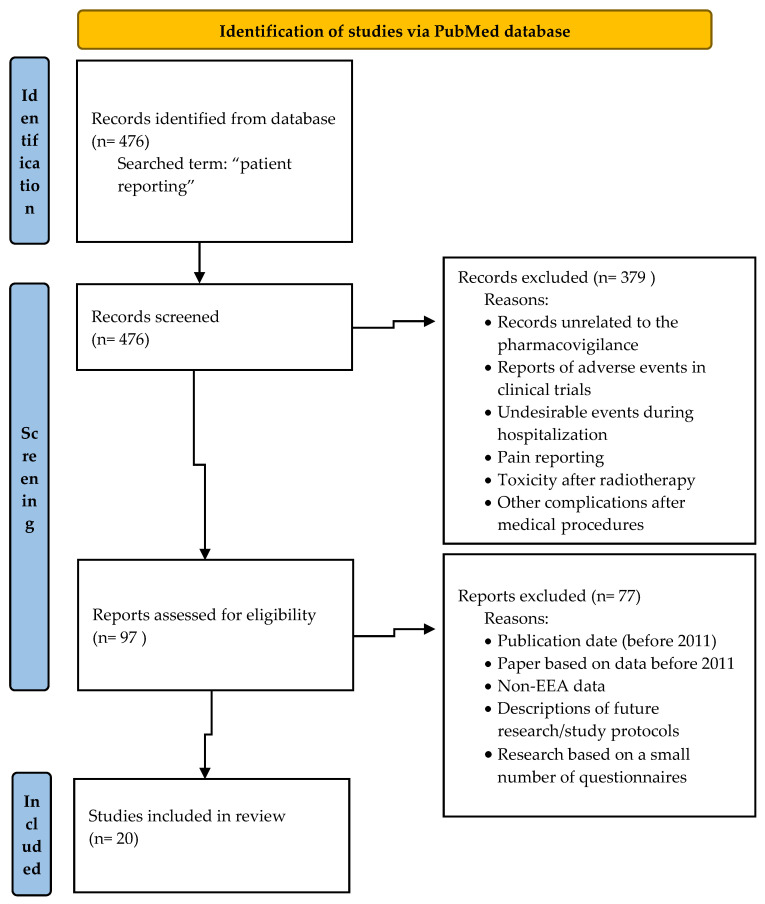
Flow diagram—identification of studies via PubMed.

To review the scientific literature, the PubMed database was searched and “patient reporting” was the search term used. The selected studies were eligible for inclusion if they addressed patients’ reporting of ADRs and were related to the role of patient reporting in pharmacovigilance or the frequency of patient reports or were focused on factors influencing patient reporting of ADRs or the methods improving patients reporting. After an analysis of 476 searched titles, 97 records were originally eligible for inclusion. Based on the analysis of articles titles/abstracts, the records unrelated to the pharmacovigilance, reports of adverse events in clinical trials, undesirable events during hospitalization, pain reporting, toxicity after radiotherapy and complications after medical procedures were excluded. The articles published before 2011 were also excluded from the study. Figure 1 presents details of inclusion and exclusion criteria for the narrative literature review.

We also collected the data related to the number of Individual Case Safety Reports (ICSRs) registered in EudraVigilance that were reported by patients, their legal representatives and caregivers, as well as those obtained from healthcare professionals related to medicines which belong to ATC code M: Musculo-skeletal system is a section of the Anatomical Therapeutic Chemical Classification System substances from the M01A Anti-inflammatory and antirheumatic products, non-steroids group. These data were collected from the www.adrreports.eu (accessed on 23 January 2021) website, which provides public access to aggregated EudraVigilance data [20]. Public access was improved in November 2017 by providing additional data available such as line listings and individual case report forms, which made it easier to detect details of reported individual case safety reports, which, in turn, enabled us to extract these data.

We analyzed the number of reported cases from 2011 until 2020 for the M01A substance group due to the fact that this group includes the medicinal products which belong to different drug availability groups e.g., prescription only, Rx drugs, as well as over-the-counter (OTC) drugs used for self-treatment. These medicines are also indicated in different populations, for long-lasting treatment and short ad hoc treatment for example in the case of pain, prescribed by a doctor or for self-treatment purposes. This group of substances was also selected because most patients are aware of potential adverse drug reactions connected with their use, which was confirmed in a population-based, non-interventional, prospective study. The study was conducted in a group of 38,928 patients who used the analyzed medicinal products, and it showed that 71% of the respondents were aware of the risk, and 60% of the patients who experienced NSAIDs-associated side effects linked it with the use of medicinal products [21].

Inclusion criteria for the described analysis are as follows:

1. Case reports sent to EudraVigilance.
2. Suspected adverse drug reaction reports for substances that belong to the M01A Anti-inflammatory and antirheumatic products, non-steroids group according to ATC code.
3. Reactions classified in all seriousness groups.
4. Reactions from EEA, non-EEA area and unspecified ones.
5. Reactions reported by healthcare professionals, non-healthcare professionals and unspecified ones.
6. Reports for all patients sex groups.
7. Reports for all patients age groups.
8. Reports for all reactions groups.
9. Reports of all reported adverse reactions.
10. Case Reports sent to EudraVigilance database between 2011 and 2020.

Table 1 and Table 2 present the data for two reporter groups separately: healthcare professionals (HP) and non-healthcare professionals (non-HP) reporter groups.

The time trend analysis was performed by means of a Joinpoint model. The method is an extended linear regression where the time trend is expressed as sections linked by the points in which the trend statistically significantly changes its direction. On the basis of the linear regression model, in which the natural logarithm of the number of ADRs for particular substances and the percentage of reports in non-HP group in all registered reports was the dependent variable, and the calendar year was the independent variable, the annual percent change (APC) and average annual percent change (AAPC) of the analyzed phenomenon were determined. To determine the statistical significance of APC and AAPC, the 95% confidence interval was determined (significance level was set at *p* = 0.05). The calculations were made with the Joinpoint Regression Program 3.5.2. (USA National Cancer Institute, Bethesda, MD, USA)—October 2011, recommended by the U.S. National Cancer Institute for this type of analysis.

## 3. Results

The analyzed scientific literature abounds in papers which zero in on the role and benefits of reports from patients and other non-HPs as well as their impact on pharmacovigilance.

The literature review by D. Prakasam et al. showed that about 11% to 35% adverse reactions reported by the patients were not identified in those which were voluntarily reported by healthcare providers, and the percentage of adverse reactions reported by patients and unregistered in medical records ranged from 5.6% to 66% [22]. P. Inácio et al. indicated that the benefits of the consumer reports were associated mainly with providing new information on the adverse reactions to medical products. Patients’ reports included more detailed descriptions of adverse events, and they referred to various drug categories and other responses in comparison with the reports by healthcare providers. Moreover, in their reports, patients described the symptoms’ severity and the impact on their everyday functioning. All that makes the obtained information on drug safety more complete and full when compared to the reports made by healthcare specialists [23]. In the analyses conducted by A.J. Avery et al. and L. Rolfes et al., a comparison was made between consumers’ reports and reports from healthcare specialists. It was proven that in comparison with healthcare specialists’ reports, the patients reported a larger number of adverse effects, and their descriptions were more detailed and indicated differences in the reported information. Patients more frequently reported reactions that affected their quality of life. Both groups reported a comparable number of cases classified as serious. It was also observed that the data on drug safety reported by both groups and analyzed jointly enabled the identification of a larger number of potential signals which were otherwise undetected when received from healthcare professionals, although their reports were more clinically informative [24,25]. Another analysis of reports on adverse reactions revealed that consumers’ reports differed in several important aspects, i.a., the range of demographic data and information on patients’ medical reports, adverse events and suspicious medications. The proportion of reports from female patients and patients with disabilities as an outcome were higher in reports provided by consumers. The data on other medications applied simultaneously were also reported more frequently by non-healthcare professionals [26].

There are some studies related to reports of adverse drug reactions by patients which are also based on querying EudraVigilance database. M. Banovac et al. also compared patient reports with those obtained from healthcare professionals. The cases reported 3 years prior to and 3 years after the date of pharmacovigilance regulations implementation were investigated. The study relied on the EudraVigilance database and showed that the number of patient reports was twice as low when the authors compared the periods before and after the date when legislation was operational. The EU members which transferred the largest number of consumer reports to EudraVigilance database included: the Netherlands, the UK, Germany, France and Italy. Patients were more willing to submit reports than healthcare professionals especially for genitourinary, hormonal and reproductive indications as well as in relation to general disorders and administration site conditions. HPs more often reported the data on the reactions, which fell within the category under investigation [27]. The available data prove that indications for the use and types of reported reactions have an impact on the patient’s involvement in the process of adverse reactions reporting. The study by L. Rolfes et al. also emphasized the increase in adverse reactions and their impact of patients’ everyday life as a factor of some significance for patients [28]. Although patient reports often require being supplemented with key information to be assessed, they provide an additional source of data for the pharmacovigilance system, and they complement the assessment made by healthcare professionals [29].

Another issue raised in the available literature concerns the potential increase of the patients engagement in pharmacovigilance in the EU. The authors’ opinions in that respect are found to be diverse. Some of them suggest that better integration of work of EU regulators should be recommended because the number of patient reports varies between the member countries. In their reported outcomes, authors underline the urgency of additional human and financial resources to be used in order to grasp the benefits of patients reporting, as now their involvement is far from a desired level [30].The study which compared the tools used in promotion of patients engagement in pharmacovigilance in different European countries revealed that the webpage of National Competent Authority had been the most popular source of information on patients reports. Other sources indicated by the authors included leaflets, posters and social media websites. The analysis also demonstrated significant differences in the number of received reports between the countries. The results indicate that the countries with less experience in direct patient reporting of adverse reactions within pharmacovigilance systems should organize promotion campaigns to encourage patients to report adverse reactions and to instruct them how to do it [31]. G. Defer et al. indicated that the potential of mobile applications and other tools for ADRs reporting could be a significant factor that may increase patient engagement in reporting adverse reactions. This can be implemented by promotion and training in tools usage and testing their effectiveness [32]. By emphasizing and explaining the significance of patient reports in the process of signal detection, the impact of patient reporting in pharmacotherapy safety monitoring may be enhanced [33]. Reports submitted by patients play a vital role in signal detection mainly in the scope of generic and psychiatric drug safety [34]. Consumer reporting constitutes a supplement to the information reported by HPs [35]. The available literature also describes future steps that could be taken with a view to increasing the impact of consumer reports in the process of pharmacotherapy safety monitoring. Nonetheless, a considerable difference is also recorded between the identified possibilities for patient reporting and their actual involvement. The promotion of patient reporting is also a potential intervention to develop public trust in pharmacovigilance activities carried out by competent authorities, and it creates a chance for the public to perceive their actual role in the process [36]. Most patients are motivated by their willingness to share the information about their cases and thereby reducing the incidence of similar reactions in other patients. However, many patients remain unaware of the possibilities of reporting such data, and others are disorientated and need assistance in that respect. The available literature also shows that the number of patient reports can be increased on condition that we raise their awareness of the opportunities of independent reporting and simplify the rules of the report submission [37]. Some authors indicate another contributing factor, i.e., the involvement of patients organizations which can encourage patients to talk to doctors or pharmacists about the suspected adverse reactions and opportunities to receive care if side effects possibly occur. The major hurdles in creating patients’ awareness of their role in pharmacovigilance include the shortage of resources, funds and insufficient involvement of competent national bodies [38]. The collaboration with other non-public organizations may create new possibilities of promotion of consumer reports, which are particularly significant as they provide additional information of drug safety independently of the data provided by sources with medical expertise. Alas, promotion of pharmacovigilance is both time- and resource-consuming, and thus government agencies opt for information campaigns, for instance via websites of agencies or by complementing the drug package leaflets with the instructions where and how to report [39]. The indices of investment in healthcare have been specified as the socio-demographic and economic factors which designated the countries with a higher index of patient reports. The factors linked to investments in healthcare were recorded to correlate with elevated patient engagement in adverse reactions reporting whereas the aspects of pharmacovigilance did not correspond directly to the increase in patients participation in drug-safety monitoring [40]. Easily accessible and user-friendly systems for patient reporting as well as innovative assessment algorithms have been indicated as future directions for the changes and development of pharmacovigilance [41]. L. Rolfes et al. investigated the level of satisfaction with the feedback received after providing the reports, and they demonstrated that the patients expected the feedback regardless of whether it was provided as a personalized letter or an automated response that confirmed the report submission [42]. According to the researchers, the feedback the patients receive may lead to an increase in their engagement in the process of medicinal products’ safety monitoring and thereby building up their trust in this type of activity.

To assess the role of non-healthcare professionals in pharmacovigilance, we analyzed a number of reports submitted by patients and their representatives and calculated the share of these reports in all reports received during the period under review.

The data reported between 2011 and 2020 to the EudraVigilance database for analyzed substances and different reporter groups are presented in Table 1 and Table 2.

### 3.1. Data on Healthcare Professionals (HP) Group

In 2011–2020 in the analyzed group, a relevant increase in the total of adverse reaction reports for all the substances under review was observed (APC = 5.02*). When analyzing individual groups of substances, an upward trend in an absolute number of reports submitted by healthcare specialists over the entire analyzed period was detected for: Naproxen APC = 10.18*; Dexketoprofen APC = 12.03*; Celecoxib APC = 4.62*; Etoricoxib APC = 7.16*. The observed rise was relevant for all the above-mentioned substances; however, the growth rate assessed by annual percent change indicated its diverse dynamics. The investigated substances also included the types whose trend in the observed period was not so unequivocal. The data concerning Ibuprofen, for instance, also demonstrated an increase in the recorded reports; however, it was relevant only in the initial period of the analysis, i.e., from 2011 to 2013, where APC = 31.47*, not from 2014 (APC = 2.90, *p* > 0.05). It should be emphasized that the year 2017 was a crucial point in the period under review, as it was in November 2017 that the obligation to report both serious adverse reactions and non-serious adverse reactions to the EudraVigilance database was implemented, which elevated the number of reports relevant, as a consequence. A relevant change in the trend of the overall number of reports made by healthcare professionals, which may result from the obligation to report also non-serious reactions to the database, was recorded for Lornoxicam, whose reports decreased relevantly since 2016 (APC = −16.33*), but increased since 2017 (APC = 3.76), albeit irrelevantly.

For some of the analyzed substances, a decrease in the number of reports submitted by healthcare professionals was noted during the period under examination and this concerned Parecoxib, Nabumetone, Dexibuprofen and Phenylbutazone. A relevant change was detected exclusively for the last of the above medications, and its APC amounted to −14.21*.

It should be underlined here that in the case of some substances, a decrease in an absolute number of reports or a lack of a marked increase in reports may be the consequence of the fall in the sales of products containing a specific substance which, in turn, may result from its diminished therapeutic significance or its replacement with other medicinal products.

**Figure 2:**
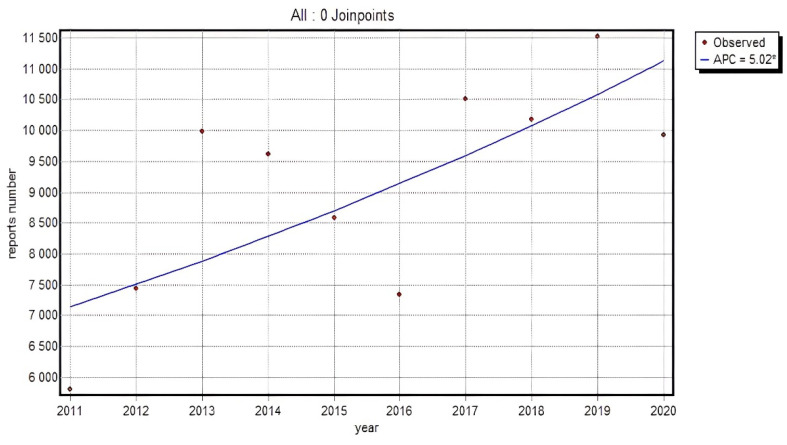
The dynamics of an absolute number of HP reports for all analyzed substances falling in the category of non-steroidal anti-inflammatory drugs; Annual Percent Change (APC).

Figure 2 presents the dynamics of the changes in an absolute number of HP reports for all analyzed substances falling in the category of non-steroidal anti-inflammatory drugs (95% CI: 0.9–9.3).

### 3.2. Data on Non-Healthcare Professionals (Non-HP) Group

An analogical analysis was performed for consumer reports in a population of non-medical specialists. An upward trend was registered for 16 substances, 4 of which showed relevance. Mean annual growth rate was varied and amounted to Indometacin APC = 29.87*; Aceclofenac APC = 31.78*; Meloxicam APC = 19.85* and Ketoprofen APC = 17.76*. The group of substances with an elevated number of consumer reports included also the ones where the trend slowed down; however, no relevant alterations were observed.

The trend appeared quite specific in Piroxicam and Naproxen and displayed greater dynamics till 2013 with a slowdown in the following years, which may have resulted from the introduced legislation which allowed the reports submission by patients and their caregivers. The same specificity of the trend was also noted in a pooled analysis of all the substances, where a relevant rise in an absolute number of submitted reports was recorded by 2013: APC = 222.91*, whereas since 2014 the growth dynamics decreased, and APC equaled 3.08 (*p* > 0.05).

**Figure 3:**
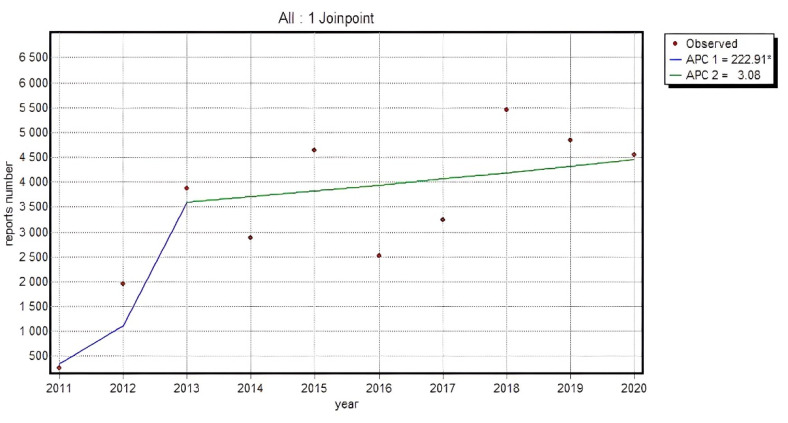
The dynamics of an absolute number of reports made by non-HP for all analyzed substances in the NSAID category; Annual Percent Change 1 (APC1), Annual Percent Change 2 (APC2).

Figure 3 presents the dynamics of an absolute number of reports made by non-HPs for all analyzed substances in the NSAID category (95% CI for APC1: 22.2–753.2; 95% CI for APC2: −9.5–17.4; 95% CI for AAPC: −9.5–59.3).

### 3.3. The Percentage of Consumer Reports in All Submitted Reports on Adverse Reactions

Since the statistical analysis of an absolute value concerning the reports submitted in particular groups provides only indicative information on the dynamics of the phenomenon, the percentage of consumer reports in a total number of reports as well as for particular substances was also investigated. The evaluation of the total number of reports from patients and their caregivers for a particular analyzed group of substances indicated a relevant increase in the percentage of a total number of reports on adverse events in the group of patients and caregivers in 2011–2013 (APC = 129.94*), and afterwards the dynamics slowed down considerably, showing a minor, irrelevant, upward trend up to the final point of analyses (APC = 0.35). The situation was slightly more diverse in the groups of particular substances. The trend progression showed one-direction changes or a slow-down in the period under review.

Relevance in the percentage of reports in non-HP groups throughout the entire investigated period was noticed for 9 substances. The highest percentage increase was identified for Flurbiprofen (38.6 percentage points APC = 32.35*), Diclofenac (with maximum 41.5 percentage points increase APC = 16.86*) and Etoricoxib (increase by 29.2 percentage points, APC = 18.64*). A similar increase (approx. 20 percentage points) was recorded for Nimesulide (APC = 15.49*), Meloxicam (APC = 10.11*) and Aceclofenac (APC = 18.08*); an upward trend was also observed for Lornoxicam (with maximum 28.9 percentage points increase, APC = 22.70*), Piroxicam (with maximum 21.6 percentage points increase, APC = 7.33*), whose peak value was recorded in 2018 (30.5%), and Ketoprofen (increased by 15.9 percentage points, APC = 13.35*). It can therefore be argued that for these substances both the legislative change, which became operational in 2012, and the obligations imposed in 2017 led to a relevant rise in the percentage of non-HP reports with regard to all the reports submitted.

For another 4 substances the trend of consumer reports percentage in a total of all the reports was less clear-cut. Naproxen was one of such substances. The year 2013 was a breakthrough point by which the percentage of non-HP reports had increased by approx. 40 percentage points (APC = 189.36*), and next it started to fall insignificantly (*p* > 0.05, APC = −4.09), with its highest value of 68.7% recorded in 2018. A similar pattern was observed for Ibuprofen with an APC reaching 144.62* (*p* < 0.05) and −2.51 (*p* > 0.05), respectively. The same tendency was observed for Celecoxib, when the reports percentage increased relevantly(APC = 283.15*) in 2011–2013 and then decreased irrelevantly to APC = −8.57. On the basis of Naproxen, Ibuprofen and Celecoxib, it can be concluded that the opportunity of adverse reactions reporting given to patients in 2012 caused an increase in the number of patient reports with regard to all the reports, while the obligation to report non-serious adverse events introduced in 2017 did not affect reporting frequency in a significant way, particularly in non-healthcare professionals. The proportion of submitted reports, however, indicates that they were made slightly more frequently by medical specialists.

**Figure 4:**
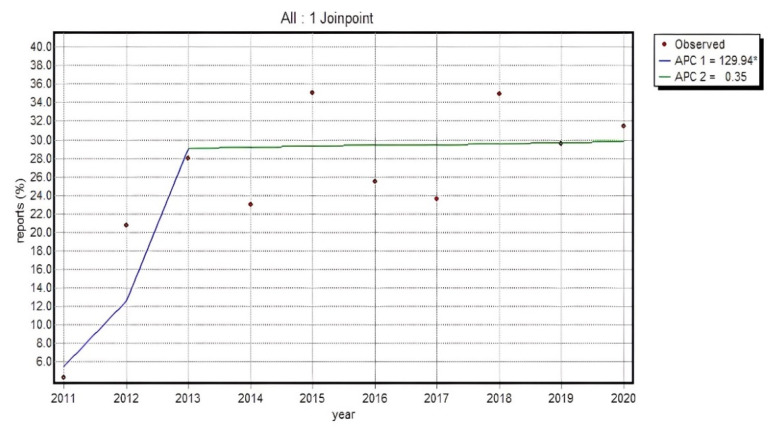
% of non-HP reports in a total of all the reports for analyzed substances in the NSAID category Annual Percent Change 1 (APC1), Annual Percent Change 2 (APC2).

It is noteworthy that the percentage of non-HP reports for all analyzed substances from non-steroidal anti-inflammatory drugs (Figure 4) increased relevantly until 2013, which confirms that the opportunity given to patients to report adverse reactions independently caused a relevant increase in the total number of reports submitted to EudraVigilance (95% CI for APC1: 20.1–340.3; 95% CI for APC2: −8.0–9.4; 95% CI for AAPC: −8.0–36.2).

The proportion of reports from patients and their caregivers was found to have risen by approx. 24% between 2011 and 2013. The most elevated values for consumer reports and the analyzed group of drugs were recorded in 2015 and 2018 (in both cases approx. 35% of all the reports). Our data analysis did not prove that the obligation to report non-serious reactions implemented in 2017 increased the percentage of non-HP reports in the total volume of reports in a relevant manner. This proves that, as a percentage, patients did not report non-serious adverse drug reactions more frequently than healthcare professionals.

## 4. Discussion

We performed a quantitative study based on measuring the number of patient reports in relation to changing legal obligations and by calculating the participation of patient reports in all adverse drug reactions reported to EV for the analyzed substance group.

The participation of non-HP reports for all analyzed substance groups relevantly increased until 2013, which confirms that allowing patients to independently submit reports increased the total number of reports in the EudraVigilance database. The percentage of reports from patients and their caregivers increased by approx. 24% between 2011 and 2013. The highest percentages of consumer reports for the analyzed group of substances were recorded in 2015 and 2018 (in both cases approx. 35% of all reports).

The analysis of the collected data did not show an increase in the percentage of non-HP reports in connection with the introduced obligation to report also non-serious adverse drug reactions. This confirms that non-healthcare professionals did not report non-serious adverse reactions more often than healthcare professionals.

The overall number of non-HP reports as well as HP reports increased in the analyzed period. We noticed that it differed between substances which belong to the M01A Anti-inflammatory and antirheumatic products, non-steroids group according to Anatomical Therapeutic Chemical (ATC) Classification. The number of non-HP reports was found to be different and dependent on medicinal product type, and its indication or drug category could have an impact on patient involvement in the pharmacovigilance process. The seriousness of reported adverse reactions was not observed to have an influence on patient involvement in the reporting process between the reporters’ groups. The growth in the number of patients’ adverse reaction reports after the implementation of the above-mentioned pharmacovigilance regulations was also shown in other EudraVigilance data-base studies [28]. The Candore G. et al. study found that less than 25% of EEA reports in EudraVigilance were classified as non-serious before the date when European Union legislation mandated the submission of such reports to EudraVigilance, and more than 60% after the legislation entered into force [43].

Based on qualitative literature data, we also showed benefits from patient reporting. According to the reviewed literature, patients reporting provided additional, complementary information to healthcare professionals’ data, mostly regarding the impact on the quality of life. Scientific literature also described the factors that affected the patient reporting and future steps to strengthen their impact on pharmacovigilance.

In our opinion, understanding the changes in patient reporting and the differences between patients’ and healthcare professionals’ reports can lead to the future improvement of pharmacovigilance processes and have a greater influece that patients may exert on safety monitoring of medicines, which, in turn, can enhance the effectiveness of pharmacovigilance system in EU. Although spontaneous reporting has many benefits, it also has a number of limitations and difficulties in diagnosing adverse drug reactions. The main challenges include underreporting and bias [44]. Several limitations were also described in the field of obtaining data from patients, e.g., these data often require verification, supplementation or additional analysis of healthcare professionals [29].

In pharmacovigilance, the outcome for the patient can be reported, but it is not always known what an additional limitation of the analyzed data is. Although pharmacovigilance activities include the possibility of obtaining follow-up data, in reality, these data are often unavailable, unlike the data from controlled clinical trials. The databases based on spontaneous reporting are an additional supplement to the data from clinical trials, and when they are more extensive, the data is wider, and they give more opportunities to identify new risks or observe their changes. Databases related to pharmacovigilance are based on spontaneous reporting, and to conduct analyses based on these data, no consent of bioethical committees or patient’s consent is required. Spontaneous reporting does not require any additional financial outlays related to the preparation of the study protocol or recruiting patients or additional work for study personnel. On the other hand, such data should frequently be complemented with additional post-authorization safety studies or other pharmacovigilance activities, e.g., patient registers. To assess and better understand the risk related to many drug therapies, some registries comprising a study design including control groups or at least defined populations at risk have been initiated. The examples of such bases include: RABBIT—the German register for the long-term observation of therapy with biologics in adult patients with rheumatoid arthritis [45], the British Society for Rheumatology Biologics Register—RA [46], the Swedish biologics register (ARTIS): observations of adverse events when using biologics in clinical practice and the Spanish registry of adverse events of biological therapies in rheumatic diseases (BIOBADASER) [47].The abovementioned registries were initiated decades ago. The databases cited above contain the data from normal clinical practice and can be classified as one of the methods for post-authorization safety studies [48]. Some literature mentions the patient register as an underused resource of medicines evaluation that can be widely used for regulatory purposes [49]. Observational studies are complementary actions and are often focused on the investigation of the risks identified by PV system findings. To assess the costs related to adverse drug events’ observational studies, widely used in PV aspects, is beneficial and this aspect is not possible to be assessed through an experimental design due to limitations defined by study protocols and time limitations [15]. The above-mentioned methods of post-authorization safety evaluation (patients registers, observational studies) require the involvement of the medical personnel. In spontaneous reporting, the patient is an independent source of ADRs which can be submitted directly. A better understanding of high-risk groups of patients can help to create better prevention strategies for the treatment of related risks. This can be achieved by a design and application of innovative methods of data analysis and online signal detection tools, which can strengthen spontaneous reporting in the future [44].

## 5. Conclusions

Our review of the data collected in the EudraVigilance system showed that legislative changes which defined an obligation to report ADRs received also from non-healthcare professionals during spontaneous monitoring affected the number of all ADRs reported to the EudraVigilance in the time interval of 2012–2020. From 2011 to 2013 the participation of patients and their caregivers reports increased by approx. 24%; then from 2014 it reached around 30% of overall reported reactions every year, so patient reporting appears to play an important part in the pharmacovigilance system and provide a source of safety information throughout their use in healthcare practice. Additionally, the seriousness of reported adverse reactions was not recognized as the factor which influenced the overall number of patient reports more than those from healthcare professionals. Furthermore, according to the literature under review, patients reporting provided additional information that was not included in healthcare professional reports and played some role in signal detection, especially in some groups of drugs and in relation to some types of reported reactions.

Despite the benefits derived from patients reporting for pharmacovigilance system, a considerable amount of work is yet to be done in order to improve patient reporting of adverse drug reactions, probably mostly in the area of awareness of adverse drug reporting rights and patients’ considerable influence on the improvements in others patients’ health statuses. Additional research on the influence of the category of medicinal product, the dosage form, indications or severity of adverse reactions on the overall number of adverse reactions reports provided by patients and healthcare professionals can contribute to designing the effective promotion of patient reporting.

## Figures and Tables

**Table 1:** Data on the number of adverse drug reaction reports between 2011 and 2020 reported by Healthcare Professionals (HP) with regard to different analyzed substances.

Substance Name	2011	2012	2013	2014	2015	2016	2017	2018	2019	2020
Phenylbutazone	13	6	5	5	2	7	6	3	2	2
Indometacin	141	524	159	172	178	159	203	208	191	165
Diclofenac	1325	1904	2252	2195	1794	1401	2188	2421	2726	2162
Acemetacin	29	16	20	23	6	18	26	24	29	19
Aceclofenac	20	27	58	48	32	24	102	47	58	50
Lornoxicam	74	48	45	35	36	24	34	26	30	31
Piroxicam	104	86	125	130	100	72	119	94	122	87
Meloxicam	140	171	253	188	212	218	268	211	242	282
Naproxen	463	574	693	730	730	798	946	898	1201	1212
Ketoprofen	506	451	1067	1326	870	494	1037	868	852	632
Flurbiprofen	85	96	143	104	102	106	136	188	160	107
Ibuprofen	1477	1955	2728	2671	2604	2320	3134	3021	3262	3004
Dexibuprofen	7	9	34	27	21	15	35	26	19	18
Dexketoprofen	78	68	95	102	82	83	121	187	216	165
Mefenamic acid	89	108	118	117	66	95	158	86	121	102
Tolfenamic acid	3	0	2	0	0	1	0	0	0	0
Celecoxib	662	797	1227	892	940	891	1102	869	1175	1273
Parecoxib	25	32	31	28	61	43	46	59	50	23
Etoricoxib	304	403	525	434	457	443	591	729	845	458
Nimesulide	246	133	365	347	240	87	226	156	188	103
Nabumetone	13	34	42	34	46	34	27	48	29	31

**Table 2:** Data on the number of adverse drug reaction reports between 2011 and 2020 reported by non-healthcare professionals (non-HP) with regard to different analyzed substances.

Substance Name	2011	2012	2013	2014	2015	2016	2017	2018	2019	2020
Phenylbutazone	0	1	2	0	1	0	2	0	2	0
Indometacin	2	13	25	33	20	17	24	50	58	58
Diclofenac	76	202	762	329	1583	450	765	1189	1062	936
Acemetacin	0	0	2	0	1	0	2	5	4	5
Aceclofenac	3	1	2	5	8	4	14	10	19	15
Lornoxicam	5	3	4	8	1	4	13	13	16	9
Piroxicam	10	19	27	36	29	20	27	41	33	25
Meloxicam	25	33	44	71	70	61	72	128	105	157
Naproxen	23	352	576	641	773	642	523	1972	1035	669
Ketoprofen	15	73	144	108	86	60	130	166	162	146
Flurbiprofen	2	7	19	7	13	24	31	90	87	74
Ibuprofen	58	645	1299	767	1343	796	914	1171	1335	1304
Dexibuprofen	0	0	0	2	0	0	5	5	16	8
Dexketoprofen	0	3	7	6	6	8	11	25	40	47
Mefenamic acid	0	5	20	16	50	16	14	26	48	27
Tolfenamic acid	0	0	0	0	0	0	0	47	4	3
Celecoxib	13	541	792	749	586	310	541	287	523	731
Parecoxib	0	1	2	0	1	4	4	2	38	53
Etoricoxib	18	29	63	60	48	64	111	179	210	244
Nimesulide	12	9	75	23	20	24	32	37	28	34
Nabumetone	0	5	8	10	3	3	3	16	12	10

## Data Availability

Data is available in a publicly accessible repository. The data presented in this study are openly available in EurdaVigilance—European database of suspected adverse drug reaction reports at www.adrreports.eu (accessed on 23 January 2021).
